# Comparative Impact of Various Exercises on Circulating Irisin in Healthy Subjects: A Systematic Review and Network Meta-Analysis

**DOI:** 10.1155/2022/8235809

**Published:** 2022-07-22

**Authors:** Fatemeh Kazeminasab, Erfan Sadeghi, Alireza Afshari-Safavi

**Affiliations:** ^1^Department of Physical Education and Sport Sciences, Faculty of Human Sciences, University of Kashan, Iran; ^2^Research Consultation Center (RCC), Shiraz University of Medical Sciences, Shiraz, Iran; ^3^Department of Biostatistics and Epidemiology, Faculty of Health, North Khorasan University of Medical Sciences, Bojnourd, Iran

## Abstract

Irisin is a myokine that is secreted from skeletal muscle during exercise and increases lipid metabolism, converting white adipose tissue to brown adipose tissue. Recent studies have shown conflicting results in relation to chronic and acute exercise and irisin. The aim of this study was to evaluate the effects of chronic and acute exercise training on circulating (plasma/serum) irisin level in healthy subjects. We conducted a search of Cochrane Library, PubMed, ISI, Scopus, Embase, and Pedro up to September 2021. A random effects network meta-analysis was performed to calculate the pooled estimate of standardized mean difference (SMD) for acute and chronic exercise effects on irisin level, using Hedge's *g* statistic. Of the 16 studies included, six were acute exercise studies (175 participants). The aerobic (Hedge's *g* = 0.23; 95% CI: -0.58, 1.03) and the anaerobic exercises (Hedge's *g* = 0.12; 95% CI: -0.45, 0.70) were associated with the increased level of irisin, compared to the control. In the ten chronic exercise studies (433 participants), the resistance training was superior to anaerobic and aerobic training (*P* score = 0.632). However, comparing acute and chronic exercise studies, acute training showed the most excellent potential as the best treatment to improve the irisin level (*P* score = 0.721). This network meta-analysis showed that acute aerobic exercise has a more effect on irisin levels than acute anaerobic exercise. Also, chronic resistance training has the greatest additive effect on irisin levels compared to chronic aerobic and anaerobic training.

## 1. Introduction

Regular exercise plays a vital role in preventing chronic diseases. However, the molecular mechanisms induced by exercise training that led to health outcomes are not fully understood. In 2012, Bostrom et al. reported that exercise increased expression of the peroxisome proliferator-activated receptor-gamma coactivator 1-alpha (PGC-1*α*) and induced FNDC5 in skeletal muscle, which breaks down to irisin and is released into the bloodstream. Irisin increases the expression of UCP1 in white adipose tissue (WAT) and turns WAT into brown adipose tissue (BAT), which leads to the induction of thermogenesis, improved lipid metabolism, glucose homeostasis, and weight loss. In general, irisin secretion can demonstrate the health benefits of exercise and be suggested as a therapeutic target for obesity and type 2 diabetes [1].

While contradictory results have been reported regarding the effect of chronic exercise on circulating (plasma/serum) irisin in adults [1–5], a study by Bostrom et al. reported that ten weeks of aerobic exercise with 65% of maximal oxygen consumption (VO2max) were associated with a twofold increase in circulating irisin compared with the untrained adult. But in another research, data showed that the irisin in trained people decreased significantly compared to inactive people [6]. Evidence from another study showed that neither endurance training (aerobic exercise) nor resistance training could increase circulating irisin levels [2]. But Tsuchiya et al. reported that resistance training had a more significant effect than endurance training on improving irisin [7]. Also, in a meta-analysis study, it was reported that the concentration of circulating irisin did not change significantly after chronic exercise and was associated with a decrease in irisin [8]. One of the methodological problems of this study is the low number of preliminary studies and the combination of the randomized controlled trial (RCT) and nonrandomized controlled study (NRs) data. Another interesting point is that some studies suggest that it is better to check the amount of circulating irisin after an acute training session. The data from these studies show that the level of irisin increases after an acute training session. Numerous studies [1, 4, 9, 10] have shown that skeletal muscle Fndc5 mRNA levels reach their peak a few hours after an acute endurance exercise session and return to the baseline level after 24 h [10]. So that, the maximal concentration of plasma irisin occurred one *h* after exercise training, thereby supporting the idea that irisin is an exercise inducible myokine [11], and its increase is transient [4, 11, 12]. Because irisin is a molecule with a low half-life and high degradation rate [2], however, these conflicting results have raised serious concerns about changes in circulating irisin, chronic exercise, and acute exercise in adults. In addition, study features such as study design and type of research population [13, 14], kinds of exercises [2, 13], and sample size [4, 15] were different.

Network meta-analysis is a novel analytic approach that compares the effects of two or more kinds of treatment simultaneously in a single analysis by combining direct and indirect evidence [16]. Additionally, it allows different interventions to be ranked for a given outcome and presents the probability of each intervention's relative efficacy, which can be helpful inform clinical decision-making [16]. Given that in previous meta-analysis studies, the population is both healthy and unhealthy individuals [17], and the data from the RCT and NRs studies were combined [8], to perform a meta-analysis network, we used the results of previous RCTs that examined the effect of a variety of exercise interventions in healthy individuals. We aimed to conduct a systematic review and network meta-analysis on the effectiveness of chronic and acute exercise training (aerobic, anaerobic, and resistance) on serum/plasma circulating irisin in healthy subjects.

## 2. Methods

We followed the Preferred Reporting Items for Systematic Reviews and Meta-Analyses Network Meta-Analyses (PRISMANMA) for our study.

### 2.1. Data Sources and Search Strategies

This meta-analysis study is reported in accordance with the Preferred Reporting Items for Systematic Reviews and Meta-Analyses (PRISMA) statement [18]. The systematic search was executed using Cochrane Library, PubMed, ISI, Scopus, Embase, and Pedro from up to September 2021. The keywords “irisin” or “FNDC5” were combined, using “AND”, with the text words “exercise” or “training” or “physical activity” or “exercise training” or “training program” or “physical activity” or “treadmill exercise” or “Physical exercise,” and for population “Healthy individuals” or “Human subject” for the PubMed search, and the same search strategy was also performed for other databases (see electronic supplementary material Appendix S1). In addition, the reference lists of previous meta-analysis articles were manually searched to ensure that no relevant articles had been missed.

### 2.2. Inclusion and Exclusion Criteria

Duplicate records were removed using Endnote software. Then, the two researchers (F.K and E.S) examined the titles and abstracts of the studies independently. Studies that met the inclusion criteria were detected and reviewed by the same authors independently. Disputes were resolved by discussion or consultation with a third author if necessary. The details of the inclusion criteria were as follows: (1) the study design should be an RCT. (2) Participants must be healthy. (3) The type of exercise interventions can include aerobic, anaerobic, or resistance training. (4) Chronic exercise included at least eight weeks of exercise. (5) Studies have a control group. (6) The primary outcome is circulating (plasma/serum) irisin levels. (7) Irisin was measured with an ELISA kit. The details of the exclusion criteria were as follows: (1) participants must not be more than 60 years old. (2) Animal studies were omitted. (3) Participant with metabolic diseases, including obesity or diabetes, was excluded. (4) The NRs studies were omitted. (5) Review, meta-analysis, cross-sectional, and crossover studies were excluded. (6) The posters were removed because they had limited information. (7) Crossover studies were omitted.

### 2.3. Data Extraction

Two researchers (F.K and E.S) extracted the data independently. Studies characteristics (study population, sample size, gender, age, and body mass index (BMI)), exercise training variables (kind of exercise, intensity, total time, number of sessions, and duration of each session), and outcome variables (mean or pretest and posttest for the level of irisin) were extracted.

### 2.4. Risk of Bias Assessment

Two researchers (F.K and E.S) examined the methodological quality of each study independently based on the Cochrane Collaboration “Risk of Bias 2” Tool, which was initially designed for assessing the quality of RCTs [19]. The quality domains were randomization process, deviations from the intended interventions, missing outcome data, measurement of the outcome, and selection of the reported result. Each study was therefore identified to be as low, moderate, or high risk of bias.

### 2.5. Statistical Analyses

The netmeta package in the R software version 4.1.2 (The R Foundation for Statistical Computing, Vienna, Austria) was applied to perform network meta-analysis by combining the direct and indirect effects of various interventions. The Cochran's *Q* and *I*-square (*I*^2^) statistics were calculated to evaluate the inconsistency and heterogeneity across studies. The *I*^2^ statistic of 25%, 50%, or 75% indicates low, moderate, or high heterogeneity. A random effects network meta-analysis was used to calculate pooled estimates of standardized mean difference (SMD) using Hedge's *g* statistic with 95% confidence intervals (95% CI). Random effects model was used since there was considerable amount of heterogeneity between the studies (*I*^2^ > 50%). Besides, it has been recommended that random effects models be employed in meta-analysis in preference to fixed effects models [20]. The network structure was graphically displayed through the network plot. Pairwise comparison of the interventions was presented in the league table using standardized mean difference (SMD, 95% confidence interval). All treatments were ranked by *P* score to calculate the amount of certainty that a single treatment is better than another competing treatment. The *P* score ranges from 0 to 1, with higher scores representing a better treatment. Potential publication bias was visually investigated using funnel plot along with a modified Egger's method by Thompson and Sharp [21]. Meta regression was also applied to assess whether duration of exercise could potentially affect the irisin level.

## 3. Results

### 3.1. Description of Included Studies

**Figure 1:**
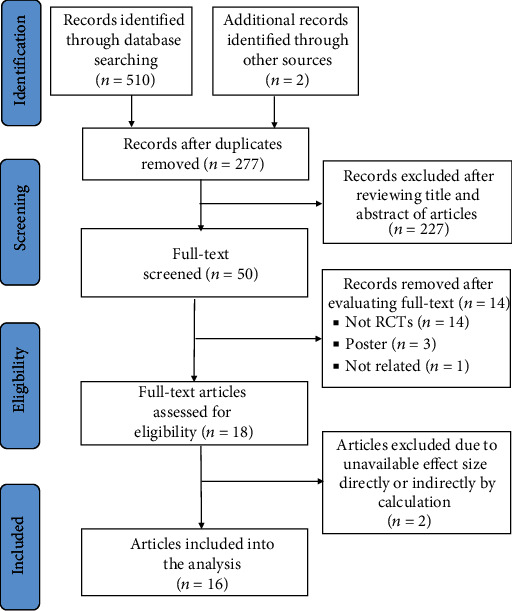
Selection of studies for inclusion. RCT: randomized controlled trial.

A total of 277 records were identified after removing duplicates. Of these records, 50 were considered potentially relevant after the preliminary screening of article titles and abstracts. Finally, after applying the inclusion and exclusion criteria, a total of 16 articles published between 2012 and 2021 with 608 participants were selected for network meta-analysis (Figure 1). The agreement rate for study selection and data extraction between the two researchers were 89.1% and 93%, respectively. In the included articles, 10 studies (433 participants) examined the effects of chronic exercise, six studies (175 participants) examined the effects of acute exercise, three studies (83 participants) examined the effects of acute aerobic training, three studies (92 participants) examined the effects of acute anaerobic training, six studies (205 participants) surveyed the impact of chronic aerobic training, five studies (212 participants) investigated the effects of chronic resistance training, and one study (16 participants) examined the effects of chronic anaerobic training. A total of 608 participants, including 360 men and 248 women, were entered in this review. The overall mean age of exercised groups and control groups were 28.9 ± 3.27 and 29.21 ± 3.63 years, respectively. Detailed study characteristics and the list of included studies are reported in Table 1.

### 3.2. Network Meta-Analysis (NMA)

**Figure 2:**
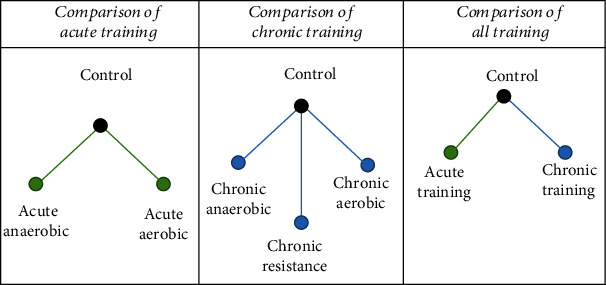
Network plots, presenting different treatment categories and direct comparisons.

We implemented three different strategies for aggregating the studies (Figure 2).

**Figure 3:**
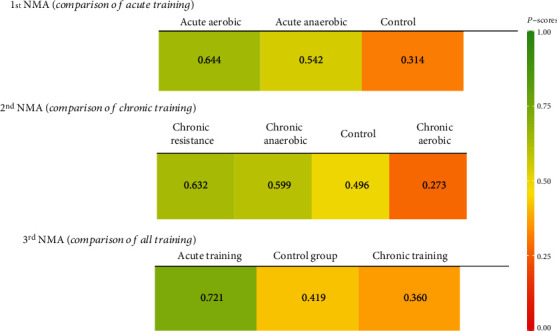
Network rank plot.

First, an NMA of irisin level was conducted based on acute training interventions, including three aerobic training studies (83 participants) and three anaerobic training studies (92 participants). The total heterogeneity was not significant among the studies (tau^2^ = 0.180, *I*^2^ = 51.1%, *Q*_total_ = 8.18; *P* = 0.085). In the direct comparisons, the acute aerobic (Hedge's *g* = 0.23; 95% CI: -0.58, 1.03) and the acute anaerobic (Hedge's *g* = 0.12; 95% CI: -0.45, 0.70) were associated with the increased level of irisin, compared with control. Based on the *P* score ranking, the acute aerobic training was superior to the acute anaerobic training and control group (*P* score = 0.644) (Figure 3). However, the league table showed no significant difference between aerobic training and anaerobic training (Hedge's *g* = 0.10; 95% CI: -0.89, 1.09) (Table 2).

In the second strategy, we implemented the NMA based on the division of chronic interventions into three groups, including aerobic training (205 participants), anaerobic training (16 participants), and resistance training (212 participants). The total heterogeneity was reported high (tau^2^ = 0.322, *I*^2^ = 71.3%, *Q*_total_ = 31.34; *P* < 0.001). The chronic aerobic (Hedge's *g* = −0.18; 95% CI: -0.73, 0.37) was associated with decreased and the chronic anaerobic (Hedge's *g* = 0.16; 95% CI: -1.12, 1.43) and the chronic resistance (Hedge's g =0.12; 95% CI: -0.47, 0.71) were associated with increased level of irisin, compared with control. Also, the chronic resistance was superior to chronic anaerobic and chronic aerobic (*P* score = 0.632) (Figure 3). However, the league table showed no significant difference between any pair of training interventions (Table 2).

Finally, we implemented an NMA based on aggregating all acute and chronic studies. The total heterogeneity was estimated at (tau^2^ = 0.221, *I*^2^ = 63.1%) and suggested that there was statistically significant heterogeneity (*Q*_total_ = 43.42; *P* < 0.001). Compared to the control, the acute interventions had a better effect on irisin level improvement with Hedge's *g* of 0.15 (95% CI: -0.35, 0.65). Still, no statistically significant. The chronic training was also associated with a nonsignificant decrease in the level of irisin (Hedge's *g* = −0.03; 95% CI: -0.37, 0.31). The acute training showed the greatest potential as the best treatment to improve the irisin level (*P* score = 0.721) (Figure 3). However, there was no evidence for a statistically difference between the acute and chronic trainings (Hedge's *g* = 0.18; 95% CI: -0.42, 0.78) (Table 2).

### 3.3. Publication Bias Analysis

**Figure 4:**
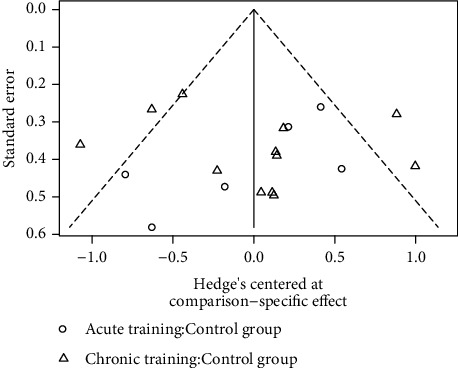
Funnel plot for evaluating the publication bias (no potential asymmetry was observed).

The funnel plot and the modified Egger's regression model (bias coefficient = −0.273, *P* = 0.860) showed no evidence of potential publication bias (Figure 4).

### 3.4. Meta Regression

The result of meta regression revealed no significant effect of exercise duration on irisin level (Table 3).

### 3.5. Risk of Bias

**Figure 5:**
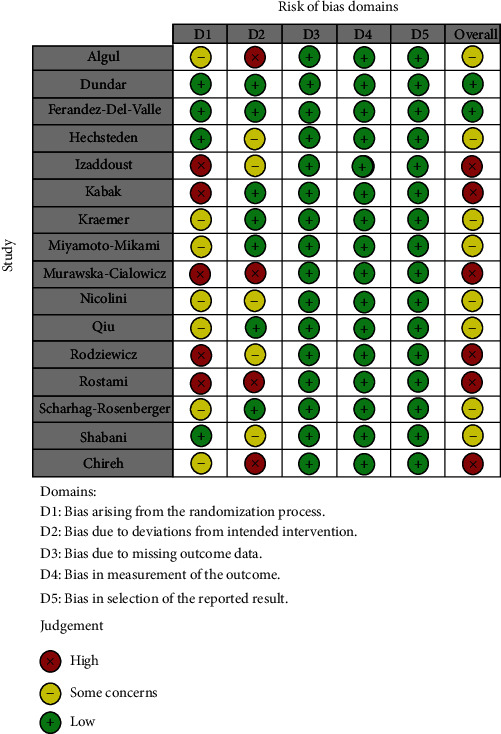
Risk of bias table.

**Figure 6:**
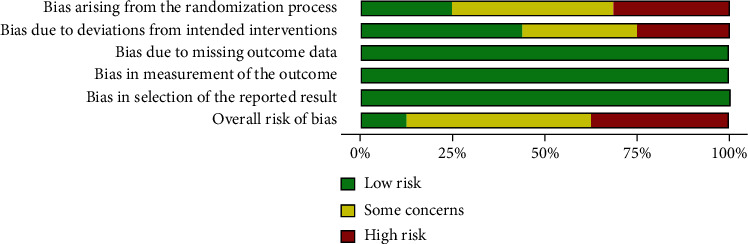
Risk of bias summary plot (percentage of studies examining the impact of exercise training in healthy subjects with low risk, some concerns, and high risk of bias for each feature of the Cochrane Collaboration “Risk of Bias 2” Tool.

Risk of bias table and summary plot were presented in Figures 5 and 6.

## 4. Discussion

### 4.1. Summary of the Main Results

The level of circulating irisin after acute aerobic and acute anaerobic exercise was higher than the control group, but no significant difference was observed between aerobic training and anaerobic training. Chronic resistance training and chronic anaerobic exercise had a moderate effect on increasing circulating irisin, but chronic aerobic exercise decreased irisin levels compared to the control group. In addition, acute exercise had a better impact on the level of irisin, but it was not significant. Also, irisin level after chronic training had a nonsignificant decrease vs. the control group. Overall, there was no significant difference between acute and chronic intervention on circulating irisin.

### 4.2. Interpretation

The impact of different forms of exercise training on plasma irisin is not clear. It is likely that chronic resistance training might be associated with an increased circulating irisin levels. First, Bostrom et al. (2012) showed that irisin increased after 10-week training healthy overweight adults [1]. Other studies have reported that circulating irisin levels increase after chronic resistance training [34, 35]. The results of the present study are consistent with the data of the Bostrom study. Second, increased muscle mass following chronic resistance training is associated with increased FNDC5 expression, and researchers in one study found that irisin was associated with muscle mass [15]. Scharhag-Rosenberger et al. showed that strength training did not change irisin levels [13]. They reported that the possible cause was the storage time of blood samples in the freezer. Serum storage in the freezer for a long time will degrade irisin, and erroneous data may be obtained.

Chronic anaerobic exercise is also associated with increased irisin levels. Huh et al. demonstrated that serum irisin correlated with exercise intensity [11]. Increased irisin may be stimulated by metabolic acidosis and the anaerobic system. For this reason, irisin has increased after chronic anaerobic exercise; however, it was not statistically significant.

On the other hand, chronic aerobic exercise has reduced plasma irisin. First, recent studies have shown that irisin is not only a myokine released from skeletal muscle but also released from adipose tissue and is known as an adipokine [36, 37]. Irisin decreases after weight loss due to surgery [15]. Given that chronic aerobic exercise is associated with weight loss [38] and body fat loss [39], it is likely that irisin in circulation after chronic aerobic exercise is reduced due to weight loss and reduce adipose tissue. Also, irisin levels also seem to be positively correlated with biceps circumference as an indicator of muscle mass [15]. Second, cross-sectional studies have also reported that irisin has a positive association with insulin resistance (IR) [40, 41] and fasting blood glucose [40, 42] in nondiabetic participants. As a result, irisin may be involved in regulating glucose homeostasis [42]. Probably, the reason for the decrease in irisin in people who have exercised chronic aerobic training is the regulation of blood glucose. Because, chronic aerobic exercise improves insulin sensitivity and decreases IR [43]. Third, recent evidence suggests that circulating irisin levels are correlated with BMI, and reduction in BMI in the trained group is associated with a decrease in irisin [15]. Fourth, irisin level reduction is associated with the pattern of decrease in the levels of lipid as a result of chronic aerobic exercise [5]. On the other hand, several studies have reported that plasma irisin increased after chronic aerobic exercise, which is consistent with the results of the study of Bostrom et al. [26, 27].

In addition, acute aerobic exercise has increased circulating irisin. Recent studies have shown that irisin increases energy intake and oxidative metabolism [44, 45]. There are conflicting results regarding exercise and energy balance, with some studies reporting that acute exercise has no effect on energy intake [46, 47]. It is noteworthy that the duration and intensity of physical activity have a significant effect on plasma irisin. As a result, future studies are needed to investigate the impact of different training protocols on energy-regulating hormones, including irisin. Also, several studies [1, 4, 9, 10] have revealed that FNDC5 mRNA levels in skeletal muscle reach their peak a one hour after an acute session of endurance exercise and return to the baseline level after 24 h [10]. Peak concentrations of irisin occurred at 0 h or 1 h after training, and that the increase is transient [4, 10–12]. As a result, the half-life of irisin in humans is less than an hour [48]. This is probably why irisin increases after acute aerobic exercise and decreases after chronic aerobic exercise. Irisin levels are usually measured immediately after an acute training, but will be calculated after chronic exercise 12 to 24 hours after the last workout. So, the short half-life of irisin can be one of the main reasons for the variation in circulating irisin levels after different exercises.

In addition, day-night rhythm affects irisin secretion. So, it has the lowest levels at 6: 00 in the mornings and highest levels at 9 in the evening [49]. Parameters such as age, gender, and BMI are also significantly associated with irisin. Researchers have reported that irisin levels are lower in women than in men, possibly due to lower muscle mass in women than men in average [50]. For this reason, in this study, participants with a mean age of 13 and 59 years and BMI between 21 and 27 were selected. And older people over 60 years and obese adults were excluded from the present study.

### 4.3. Perspective

There has been no systematic review before this one of network meta-analysis designed to evaluate the impact of various exercises on circulating irisin. Our results show that acute aerobic training and chronic resistance training increase circulating irisin level compared to acute anaerobic training and chronic aerobic and anaerobic, respectively. Also, our findings show that acute training had a better effect on irisin level improvement vs. chronic training. But more further studies with more diverse designs to compare the types of exercise (acute and chronic) in healthy and patients individuals should be performed in the future.

### 4.4. Strengths and Limitations

The present network meta-analysis has several strengths. First, the previous meta-analysis study by Qiu et al. examined three RCT studies, while the present meta-analysis evaluated 16 RCT studies with 608 subjects [8]. Second, in previous meta-analysis studies, chronic aerobic, resistance, anaerobic exercise, acute aerobic, and anaerobic training studies were not separated or compared. Third, to minimize report bias, the study methodology was registered a priori. Fourth, it is to date, the impact of chronic and acute exercise on circulating irisin have not been compared in network meta-analysis studies. Fifth, in this study, obese and diabetic individuals were excluded, and only the impact of various types of physical exercise on irisin levels in healthy individuals was investigated.

The study also has some limitations. There were few studies on the effect of acute exercise on irisin levels. Lack of studies evaluating the direct comparison of acute and chronic or aerobic and resistance interventions so that we could have a precise estimation of inconsistency between the studies. Also, a relatively small number of studies have also examined the impact of combined resistance and aerobic exercise on circulating irisin.

## 5. Conclusions

This network meta-analysis showed that the acute aerobic training was superior to the acute anaerobic training and control group. Also, chronic resistance training has the most significant additive effect on irisin levels than chronic aerobic and anaerobic training. This network meta-analysis also showed that study design in randomized controlled trials, type of exercise, and training intensity might be the primary sources for contradictory results reported in the literature.

## Figures and Tables

**Table 1:** Characteristics of the studies included in the network meta-analysis.

Study (year)	Population	Exercised group: age (years)	Control group: age (years)	Exercised group: BMI	Control group: BMI	Sex (M/F)	Description of exercise training intervention and control	Blood sample	Blood sampling time
Algul et al. (2017) [22]	Healthy young men	19.2 ± 0.7	19.5 ± 0.6	21.3 ± 0.4	21.7 ± 0.4	60 M	*K*: aerobic exercise, acute *I*: 64-76% MHR (moderate-intensity) *T*: 30 min *N*: one session	Serum	Before and immediately after exercise

Dundar et al. (2021) [5]	Healthy adolescent boys	13-16	13-16	23.75 ± 1.75	24.85 ± 1.6	34 M	*K*: aerobic exercise, chronic (basketball training) *I*: no mention *T*: 120 min *N*: 5 sessions/week *D*: 8 weeks	Serum	Before and after the eight-week exercise program

Fernandez-del-Valle et al. (2018) [23]	Healthy young adult	Males: 21.18 ± 1.93	Females: 21.35 ± 2.52	22.11 ± 1.68	23.04 ± 1.94	14 M/12 F	*K*: resistance exercise, chronic *I*: 70-80% 1RM (high-intensity) *T*: 55 min *N*: 3 sessions/week *D*: 3 weeks	Serum	Before and after the exercise

Hecksteden et al. (2013)^A^ [2]	Healthy men and women	49 ± 7	50 ± 7	23.5 ± 3.5	24.5 ± 3.1	21 M/62 F	*K*: aerobic exercise, chronic *I*: 60% heart rate reserve *T*: 45 min *N*: 3 sessions/week *D*: 26 weeks	Serum	Before the training period and at 2 to 7 days after the final training bout

Hecksteden et al. (2013)^B^ [2]	Healthy men and women	48 ± 7	50 ± 7	24.9 ± 3.4	24.5 ± 3.1	30 M/49 F	*K*: resistance exercise, chronic *I*: two sets of 15 repetitions with 100% of the 20-repetition maximum (high-intensity) *N*: 3 sessions/week *D*: 26 weeks	Serum	Before the training period and at 2 to 7 days after the final training bout

Izaddoust and Shabani (2017) [24]	Untrained women	24.6 ± 2.45	26.44 ± 4.18	23.45 ± 2.83	23.28 ± 2.62	16 F	*K*: resistance exercise, chronic *I*: 65-75% 1RM *T*: 65 min *N*: 3 sessions/week *D*: 8 weeks	Serum	Before and 24 hours the after last training session

Kabak et al. (2018) [25]	Professional kick-boxers	20.20 ± 1.62	20.00 ± 1.33	23.47 ± 2.94	24.14 ± 2.66	30 M	*K*: anaerobic exercise, acute *I*: HIIT *T*: four 30-s Wingate anaerobic test *N*: one session	Plasma	Before and after exercise (within 1 min)

Miyamoto-Mikami et al. (2015) [26]	Healthy young women and men	21 ± 1	21 ± 1	21.9 ± 1.7	23.1 ± 3.4	16 M/9 F	*K*: aerobic exercise, chronic *I*: 60-70% vo2max *T*: 55 min *N*: 3 sessions/week *D*: 8 weeks	Serum	Before and after exercise

Murawska-Cialowicz et al. (2020) [27]	Healthy men	32.39 ± 6.63	25.35 ± 3.28	25.75 ± 2.94	24.16 ± 2.19	25 M	*K*: aerobic exercise, chronic *I*: HIIT *T*: 60 min *N*: 2 sessions/week *D*: 8 weeks	Serum	Before and after the 8-week training

Nicolini et al. (2020) [28]	Healthy young men	23 ± 3	25 ± 4	No mention	No mention	40 M	*K*: anaerobic running, acute *I*: high-intensity interval exercise *T*: 17.5 min *N*: one session	Serum	Before and 30 min after exercise

Qiu et al. (2018) [29]	Healthy adults	22.0 ± 1.1	22.2 ± 1.9	22.0 ± 1.1	22.2 ± 1.9	16 M	*K*: aerobic running, acute *I*: 80% vo2max (high-intensity) *T*: 60 min *N*: one session	Serum	Before and 10 min after exercise

Rodziewicz et al. (2020) [30]	Master endurance master athletes	58.6 ± 4.3	57.4 ± 2.9	24.2 ± 0.5	24.0 ± 0.4	22 M	*K*: anaerobic running, acute *I*: high-intensity *T*: exercise test until exhaustion *N*: one session	Plasma	Before and 10 min after exercise

Rostami et al. (2019) [31]	Healthy young untrained women	24.66 ± 2.29	26.44 ± 4.18	26.7 ± 2.7	23.28 ± 2.62	20 F	*K*: aerobic exercise, chronic *I*: 65-75%MHR (moderate-intensity) *T*: 65 min *N*: 3 sessions/week *D*: 8 weeks	Serum	48 h before and 24 h after the last training session

Scharhag-Rosenberger et al. (2014) [13]	Healthy men and women	47 ± 7	50 ± 7	25 ± 3.4	24.2 ± 3.2	29 M/45 F	*K*: resistance exercise, chronic *I*: 16–20 repetitions at 64%–71% 1RM *N*: 3 sessions/week *D*: 24 weeks	Serum	Before and after exercise

Kraemer et al. (2014)^A^ [12]	Healthy men	22.71 ± 1.6	22.71 ± 1.6	24.29 ± 2.94	24.29 ± 2.94	7 M	*K*: aerobic exercise, acute *I*: 60% of VO2max (moderate-intensity) *T*: 90 min *N*: one session	Plasma	Before and 54 min after exercise

Shabani and Izaddoust (2018)^A^ [32]	Healthy untrained women	24.66 ± 2.29	25.50 ± 4.80	26.70 ± 2.70	23.28 ± 2.62	18 F	*K*: aerobic exercise, chronic *I*: 60–75% MHR *T*: 65 min *N*: 3 sessions/week *D*: 8 weeks	Serum	Before and after exercise

Shabani and Izaddoust (2018)^B^ [32]	Healthy untrained women	24.60 ± 2.45	25.50 ± 4.80	23.45 ± 2.83	23.28 ± 2.62	17 F	*K*: resistance exercise, chronic *I*: 70-75% 1RM *T*: 65 min *N*: 3 sessions/week *D*: 8 weeks	Serum	Before and after exercise

Chireh et al. (2018) [33]	Healthy men	18-28	18-28	No mention	No mention	16 M	*K*: anaerobic exercise, chronic *I*: 50% Wmax *T*: 10 min *N*: 3 sessions/week *D*: 3 weeks	Plasma	Before and 48 h after exercise

Variables presented as mean ± SD. *K*: kind of exercise; *I*: intensity of exercise; *T*: time of each session; *N*: number of sessions; *D*: duration of exercise; MHR: maximum heart rate; HIIT: high intensity interval training; H: hour.

**Table 2:** League table of NMA.

1^st^ NMA (irisin level; SMD (95% CI) *Comparison of acute training*			
*Acute aerobic*	—	0.23 [-0.58; 1.03]	
0.10 [-0.89; 1.09]	*Acute anaerobic*	0.12 [-0.45; 0.70]	
0.23 [-0.58; 1.03]	0.12 [-0.45; 0.70]	*Control*	

2^nd^ NMA (irisin level; SMD (95% CI) *Comparison of chronic training*			
*Chronic aerobic*	—	—	-0.18 [-0.73; 0.37]
-0.33 [-1.72; 1.05]	*Chronic anaerobic*	—	0.16 [-1.12; 1.43]
-0.30 [-1.11; 0.51]	0.04 [-1.37; 1.44]	*Chronic resistance*	0.12 [-0.47; 0.71]
-0.18 [-0.73; 37]	0.16 [-1.12; 1.43]	0.12 [-0.47; 0.71]	*Control*

3^rd^ NMA (irisin level; SMD (95% CI) *Comparison of all training*			
*Acute training*	—	0.15 [-0.35; 0.65]	
0.18 [-0.42; 0.78]	*Chronic training*	-0.03 [-0.37; 0.31]	
0.15 [-0.35; 0.65]	-0.03 [-0.37; 0.31]	*Control*	

**Table 3:** Meta regression model for evaluating the effect of exercise duration on irisin level (pooled Hedges SMD as the response variable).

Variable		Coefficient (S.E)	95% CI	*I* ^2^	*P*value
Duration of exercise	Acute	-0.005 (0.010)	-0.037, 0.026	56.5%	0.631
	Chronic	-0.009 (0.021)	-0.056, 0.037	70%	0.655

## Supplementary Materials

Supplementary Materials Search queries: Embase, ISI, Cochrane, PubMed, and Scopus.
