# Supplementary material for: Comparative Impact of Various Exercises on Circulating Irisin in Healthy Subjects: A Systematic Review and Network Meta-Analysis
Source: Oxid Med Cell Longev. 2022 Jul 22;2022:8235809. doi: 10.1155/2022/8235809 (PMC9337948; doi:10.1155/2022/8235809)
Supplement: Supplementary Materials — Search queries: Embase, ISI, Cochrane, PubMed, and Scopus. [file 8235809.f1.zip › cochrane.docx]

Search Name: Irisin

Date Run: 26/01/2021 02:47:17

Comment:

ID Search Hits

#1 MeSH descriptor: [Adult] explode all trees 462536

#2 (Adult*):ti,ab,kw 666954

#3 MeSH descriptor: [Adolescent] explode all trees 104026

#4 (Adolescent*):ti,ab,kw 135277

#5 (teenager*):ti,ab,kw 954

#6 MeSH descriptor: [Humanism] explode all trees 11

#7 (human*):ti,ab,kw 1054934

#8 MeSH descriptor: [Healthy Volunteers] explode all trees 3534

#9 (Healthy Volunteer*):ti,ab,kw 48068

#10 MeSH descriptor: [Healthy People Programs] explode all trees 12

#11 (Healthy People*):ti,ab,kw 6215

#12 (Healthy individual*):ti,ab,kw 18751

#13 (Human subject*):ti,ab,kw 107049

#14 (healthy):ti,ab,kw 142765

#15 {OR #1-#14} 1145527

#16 MeSH descriptor: [Exercise] explode all trees 24678

#17 (Exercise):ti,ab,kw 97361

#18 (training):ti,ab,kw 91296

#19 (exercise training):ti,ab,kw 31924

#20 (training program):ti,ab,kw 28387

#21 MeSH descriptor: [Sports] explode all trees 15629

#22 (sport*):ti,ab,kw 9674

#23 (physical activity):ti,ab,kw 41666

#24 (treadmill exercise):ti,ab,kw 6721

#25 (Physical exercise):ti,ab,kw 38454

#26 MeSH descriptor: [Endurance Training] explode all trees 59

#27 (Endurance Training):ti,ab,kw 6014

#28 (aerobic):ti,ab,kw 15486

#29 (Aerobic workout):ti,ab,kw 115

#30 MeSH descriptor: [Resistance Training] explode all trees 3454

#31 (Resistance Training):ti,ab,kw 12372

#32 (strength workout):ti,ab,kw 177

#33 MeSH descriptor: [Circuit-Based Exercise] explode all trees 27

#34 (combined exercise):ti,ab,kw 9076

#35 (Chronic exercise):ti,ab,kw 14688

#36 (acute exercise):ti,ab,kw 8661

#37 {OR #16-#36} 184001

#38 MeSH descriptor: [Control Groups] explode all trees 111

#39 (Control group*):ti,ab,kw 334983

#40 MeSH descriptor: [Volunteers] explode all trees 3829

#41 (Volunteers):ti,ab,kw 64617

#42 ("not trained"):ti,ab,kw 156

#43 (untrained):ti,ab,kw 2276

#44 (sedentary):ti,ab,kw 7635

#45 (unexercised):ti,ab,kw 5

#46 {OR #38-#45} 396510

#47 (FNDC5 protein, human):ti,ab,kw 10

#48 (Irisin):ti,ab,kw 185

#49 (irisin level):ti,ab,kw 89

#50 (blood irisin):ti,ab,kw 123

#51 (plasma irisin):ti,ab,kw 34

#52 (serum irisin):ti,ab,kw 92

#53 (FNDC5):ti,ab,kw 15

#54 {OR #47-#53} 186

#55 #15 AND #37 AND #46 AND #54 47
