# Supplementary material for: Comparative Impact of Various Exercises on Circulating Irisin in Healthy Subjects: A Systematic Review and Network Meta-Analysis
Source: Oxid Med Cell Longev. 2022 Jul 22;2022:8235809. doi: 10.1155/2022/8235809 (PMC9337948; doi:10.1155/2022/8235809)
Supplement: Supplementary Materials — Search queries: Embase, ISI, Cochrane, PubMed, and Scopus. [file 8235809.f1.zip › Embase.docx]

No. Query Results Date

#54 #9 AND #30 AND #36 AND #40 AND #53 132 25-Jan-21

#53 #41 OR #42 OR #43 OR #44 OR #45 OR #46 OR #47 OR #48 OR #49 OR #50 OR #51 OR #52 10073137 25-Jan-21

#52 "'experimental study'/exp OR 'experimental stud*':ab,ti OR 'experimental':ab,ti" 1267134 25-Jan-21

#51 "'nonrandomized controlled trial':ab,ti OR 'non-randomized controlled trial':ab,ti OR 'nonrandomized':ab,ti OR 'non-randomized stud*':ab,ti" 30511 25-Jan-21

#50 "'group'/exp OR 'group*':ab,ti" 5381718 25-Jan-21

#49 "'intervention'/exp OR 'intervention':ab,ti" 896020 25-Jan-21

#48 "'placebo'/exp OR 'placebo':ab,ti" 470992 25-Jan-21

#47 'drug therapy'/exp 2960087 25-Jan-21

#46 "'randomization'/exp OR 'randomiz*':ab,ti OR randomly:ab,ti" 1220067 25-Jan-21

#45 "'clinical trial':ab,ti OR rct:ab,ti OR trial:ab,ti" 927517 25-Jan-21

#44 'controlled clinical trial'/exp OR 'controlled clinical trial (topic)'/exp 1012126 25-Jan-21

#43 'randomized controlled trial'/exp 641569 25-Jan-21

#42 'randomized controlled trial (topic)'/exp 194607 25-Jan-21

#41 'clinical trial (topic)'/exp 343221 25-Jan-21

#40 #37 OR #38 OR #39 1918 25-Jan-21

#39 "'irisin level':ab,ti OR 'blood irisin':ab,ti OR 'plasma irisin':ab,ti OR 'serum irisin':ab,ti" 589 25-Jan-21

#38 "'irisin'/exp OR 'irisin':ab,ti" 1847 25-Jan-21

#37 "'fndc5 protein human'/exp OR 'fndc5':ab,ti" 510 25-Jan-21

#36 #31 OR #32 OR #33 OR #34 OR #35 1002339 25-Jan-21

#35 "unexercised:ab,ti" 191 25-Jan-21

#34 "'sedentary lifestyle'/exp OR 'sedentary':ab,ti" 47522 25-Jan-21

#33 "'not trained':ab,ti OR untrained:ab,ti" 14085 25-Jan-21

#32 "'volunteer'/exp OR 'volunteer*':ab,ti" 274206 25-Jan-21

#31 "'control group'/exp OR 'control group*':ab,ti" 691870 25-Jan-21

#30 #10 OR #11 OR #12 OR #13 OR #14 OR #15 OR #16 OR #17 OR #18 OR #19 OR #20 OR #21 OR #22 OR #23 OR #24 OR #25 OR #26 OR #27 OR #28 OR #29 1830307 25-Jan-21

#29 "'acute exercise'/exp OR 'acute exercise':ab,ti" 3815 25-Jan-21

#28 "'chronic exercise'/exp OR 'chronic exercise':ab,ti" 1547 25-Jan-21

#27 "'combined exercise':ab,ti" 845 25-Jan-21

#26 'circuit training'/exp 247 25-Jan-21

#25 "strength:ab,ti OR 'strength workout':ab,ti" 355296 25-Jan-21

#24 "'resistance training'/exp OR 'resistance training':ab,ti" 22540 25-Jan-21

#23 "'aerobic workout':ab,ti" 30 25-Jan-21

#22 "'aerobic exercise'/exp OR 'aerobic':ab,ti" 110683 25-Jan-21

#21 "'endurance training'/exp OR 'endurance training':ab,ti" 10883 25-Jan-21

#20 "'physical exercise':ab,ti" 22286 25-Jan-21

#19 "'treadmill exercise'/exp OR 'treadmill exercise':ab,ti" 22905 25-Jan-21

#18 "'physical activity'/exp OR 'physical activity':ab,ti" 489105 25-Jan-21

#17 "'sport'/exp OR sport*:ab,ti" 231919 25-Jan-21

#16 "'training programs':ab,ti" 21966 25-Jan-21

#15 'training programs'/exp 903 25-Jan-21

#14 "'exercise training':ab,ti" 23644 25-Jan-21

#13 "'training':ab,ti" 577543 25-Jan-21

#12 'training'/exp 90152 25-Jan-21

#11 "'exercise':ab,ti" 362274 25-Jan-21

#10 'exercise'/exp 366526 25-Jan-21

#9 #1 OR #2 OR #3 OR #4 OR #5 OR #6 OR #7 OR #8 24479011 25-Jan-21

#8 "healthy:ab,ti" 1195396 25-Jan-21

#7 "'human subject*':ab,ti" 38681 25-Jan-21

#6 "'normal human'/exp OR 'healthy people':ab,ti OR 'healthy individual*':ab,ti OR 'healthy lifestyle'/exp" 833630 25-Jan-21

#5 "'normal human'/exp OR 'healthy people':ab,ti" 767141 25-Jan-21

#4 "'human'/exp OR human*:ab,ti" 23742974 25-Jan-21

#3 "teenager*:ab,ti" 21009 25-Jan-21

#2 "'adolescent'/exp OR adolescent*:ab,ti" 1780600 25-Jan-21

#1 "'adult'/exp OR 'adult*':ab,ti" 9807919 25-Jan-21
