# Supplementary material for: Comparative Impact of Various Exercises on Circulating Irisin in Healthy Subjects: A Systematic Review and Network Meta-Analysis
Source: Oxid Med Cell Longev. 2022 Jul 22;2022:8235809. doi: 10.1155/2022/8235809 (PMC9337948; doi:10.1155/2022/8235809)
Supplement: Supplementary Materials — Search queries: Embase, ISI, Cochrane, PubMed, and Scopus. [file 8235809.f1.zip › ISI.docx]

| # 6 | [**93**](https://apps.webofknowledge.com/summary.do?product=WOS&doc=1&qid=16&SID=E1Gs5jIJDwd1icNm6nh&search_mode=CombineSearches&update_back2search_link_param=yes) | #5 AND #4 AND #3 AND #2 AND #1  *Indexes=SCI-EXPANDED, SSCI, A&HCI, CPCI-S, CPCI-SSH, BKCI-S, BKCI-SSH, ESCI, CCR-EXPANDED, IC Timespan=1900-2021* |
| --- | --- | --- |
| # 5 | [**7,843,580**](https://apps.webofknowledge.com/summary.do?product=WOS&doc=1&qid=15&SID=E1Gs5jIJDwd1icNm6nh&search_mode=AdvancedSearch&update_back2search_link_param=yes) | (TS=(“clinical trials”) OR TS=(“randomized controlled trial”) OR TS=(“controlled clinical trial”) OR TS=(“clinical trial”) OR TS=(“randomized”) OR TS=(“placebo”) OR TS=("drug therapy") OR TS= (“randomly”) OR TS= (“trial”) OR TS= (“groups”) OR TS=(“intervention”) OR TS=(RCT) OR TS=(Non-randomized controlled trials) OR TS=(non-randomized) OR TS=(experimental study) OR TS=(experimental) OR TS=(non-randomized stud*))  *Indexes=SCI-EXPANDED, SSCI, A&HCI, CPCI-S, CPCI-SSH, BKCI-S, BKCI-SSH, ESCI, CCR-EXPANDED, IC Timespan=1900-2021* |
| # 4 | [**1,623**](https://apps.webofknowledge.com/summary.do?product=WOS&doc=1&qid=14&SID=E1Gs5jIJDwd1icNm6nh&search_mode=AdvancedSearch&update_back2search_link_param=yes) | (TS=(Irisin) OR TS=(Irisin level) OR TS=(blood Irisin) OR TS=(plasma Irisin) OR TS=(serum Irisin) OR TS=(FNDC5))  *Indexes=SCI-EXPANDED, SSCI, A&HCI, CPCI-S, CPCI-SSH, BKCI-S, BKCI-SSH, ESCI, CCR-EXPANDED, IC Timespan=1900-2021* |
| # 3 | [**1,508,722**](https://apps.webofknowledge.com/summary.do?product=WOS&doc=1&qid=12&SID=E1Gs5jIJDwd1icNm6nh&search_mode=AdvancedSearch&update_back2search_link_param=yes) | (TS=(Control Groups) OR TS=(Control Group*) OR TS=(Volunteers) OR TS=(“not trained”) OR TS=(untrained) OR TS=(sedentary) OR TS=(unexercised))  *Indexes=SCI-EXPANDED, SSCI, A&HCI, CPCI-S, CPCI-SSH, BKCI-S, BKCI-SSH, ESCI, CCR-EXPANDED, IC Timespan=1900-2021* |
| # 2 | [**3,039,613**](https://apps.webofknowledge.com/summary.do?product=WOS&doc=1&qid=11&SID=E1Gs5jIJDwd1icNm6nh&search_mode=AdvancedSearch&update_back2search_link_param=yes) | (TS=(Exercise) OR TS=(training) OR TS=(exercise training) OR TS=(training program) OR TS=(sport) OR TS=(sports) OR TS=(physical activity) OR TS=(treadmill exercise) OR TS=(Physical exercise) OR TS=(Endurance Training) OR TS=(aerobic) OR TS=(Aerobic workout) OR TS=(Resistance Training) OR TS=(strength) OR TS=(strength workout) OR TS=(Circuit-Based Exercise) OR TS=(combined exercise) OR TS=(Chronic exercise) OR TS=(acute exercise))  *Indexes=SCI-EXPANDED, SSCI, A&HCI, CPCI-S, CPCI-SSH, BKCI-S, BKCI-SSH, ESCI, CCR-EXPANDED, IC Timespan=1900-2021* |
| # 1 | [**6,565,479**](https://apps.webofknowledge.com/summary.do?product=WOS&doc=1&qid=10&SID=E1Gs5jIJDwd1icNm6nh&search_mode=AdvancedSearch&update_back2search_link_param=yes) | (TS=(Adult) OR TS=(Adult*) OR TS=(Adolescent) OR TS=(Adolescent*) OR TS=(teenager*) OR TS=(humans) OR TS=(Healthy Volunteers) OR TS=(Healthy People Programs) OR TS=(Healthy individuals) OR TS=(Human subject) OR TS=(healthy))  *Indexes=SCI-EXPANDED, SSCI, A&HCI, CPCI-S, CPCI-SSH, BKCI-S, BKCI-SSH, ESCI, CCR-EXPANDED, IC Timespan=1900-2021* |
