# Supplementary material for: Comparative Impact of Various Exercises on Circulating Irisin in Healthy Subjects: A Systematic Review and Network Meta-Analysis
Source: Oxid Med Cell Longev. 2022 Jul 22;2022:8235809. doi: 10.1155/2022/8235809 (PMC9337948; doi:10.1155/2022/8235809)
Supplement: Supplementary Materials — Search queries: Embase, ISI, Cochrane, PubMed, and Scopus. [file 8235809.f1.zip › PUBMED.docx]

Search: (((((((((Adult[MeSH Terms]) OR (Adult*[Title/Abstract])) OR (Adolescent[MeSH Terms])) OR (Adolescent*[Title/Abstract])) OR (teenager*[Title/Abstract])) OR ((((((humans[MeSH Terms]) OR (Healthy Volunteers[MeSH Terms])) OR (Healthy People Programs[MeSH Terms])) OR (Healthy individuals[Title/Abstract])) OR (Human subject[Title/Abstract])) OR (healthy[Title/Abstract]))) AND (((((((((((((((((((((((Exercise[MeSH Terms]) OR (Exercise[Title/Abstract])) OR (training[Title/Abstract])) OR (exercise training[Title/Abstract])) OR (training program[Title/Abstract])) OR (sports[MeSH Terms])) OR (sport[Title/Abstract])) OR (physical activity[Title/Abstract])) OR (treadmill exercise[Title/Abstract])) OR (Physical exercise[Title/Abstract]))) OR (Endurance Training[MeSH Terms])) OR (Endurance Training[Title/Abstract])) OR (aerobic[Title/Abstract])) OR (Aerobic workout[Title/Abstract])) OR (Resistance Training[MeSH Terms])) OR (Resistance Training[Title/Abstract])) OR (strength[Title/Abstract])) OR (strength workout[Title/Abstract])) OR (Circuit-Based Exercise[MeSH Terms])) OR (combined exercise[Title/Abstract])) OR (Chronic exercise[Title/Abstract])) OR (acute exercise[Title/Abstract]))) AND (((((((Control Groups[MeSH Terms]) OR (Control group*[Title/Abstract])) OR (Volunteers[MeSH Terms])) OR ("not trained"[Title/Abstract])) OR (untrained[Title/Abstract])) OR (sedentary[Title/Abstract])) OR (unexercised[Title/Abstract]))) AND (((((((FNDC5 protein, human [Supplementary Concept]) OR (Irisin[Title/Abstract])) OR (irisin level[Title/Abstract])) OR (blood irisin[Title/Abstract])) OR (plasma irisin[Title/Abstract])) OR (serum irisin[Title/Abstract])) OR (FNDC5[Title/Abstract]))) AND ((((((("clinical trials as topic"[MeSH Terms] OR "randomized controlled trial"[Publication Type] OR "controlled clinical trial"[Publication Type] OR "clinical trial"[Publication Type] OR "randomized"[Title/Abstract] OR "placebo"[Title/Abstract] OR "drug therapy"[MeSH Subheading] OR "randomly"[Title/Abstract] OR "trial"[Title] OR "groups"[Title/Abstract] OR "intervention"[Title/Abstract]) OR (rct)) OR (Non-Randomized Controlled Trials as Topic[MeSH Terms])) OR (non randomized[Title/Abstract])) OR (experimental study[Title/Abstract])) OR (experimental[Title/Abstract])) OR (non-randomized stud*[Title/Abstract]))
