# Supplementary material for: Comparative Impact of Various Exercises on Circulating Irisin in Healthy Subjects: A Systematic Review and Network Meta-Analysis
Source: Oxid Med Cell Longev. 2022 Jul 22;2022:8235809. doi: 10.1155/2022/8235809 (PMC9337948; doi:10.1155/2022/8235809)
Supplement: Supplementary Materials — Search queries: Embase, ISI, Cochrane, PubMed, and Scopus. [file 8235809.f1.zip › Scopus.docx]

| 6 | ( ( TITLE-ABS-KEY ( adult )  OR  TITLE-ABS-KEY ( adult* )  OR  TITLE-ABS-KEY ( adolescent )  OR  TITLE-ABS-KEY ( adolescent* )  OR  TITLE-ABS-KEY ( teenager* )  OR  TITLE-ABS-KEY ( humans )  OR  TITLE-ABS-KEY ( healthy  AND volunteers )  OR  TITLE-ABS-KEY ( healthy  AND people  AND programs )  OR  TITLE-ABS-KEY ( healthy  AND individuals )  OR  TITLE-ABS-KEY ( human  AND subject )  OR  TITLE-ABS-KEY ( healthy ) ) )  AND  ( ( TITLE-ABS-KEY ( exercise )  OR  TITLE-ABS-KEY ( training )  OR  TITLE-ABS-KEY ( exercise  AND training )  OR  TITLE-ABS-KEY ( training  AND program )  OR  TITLE-ABS-KEY ( sport )  OR  TITLE-ABS-KEY ( sports )  OR  TITLE-ABS-KEY ( physical  AND activity )  OR  TITLE-ABS-KEY ( treadmill  AND exercise )  OR  TITLE-ABS-KEY ( physical  AND exercise )  OR  TITLE-ABS-KEY ( endurance  AND training )  OR  TITLE-ABS-KEY ( aerobic )  OR  TITLE-ABS-KEY ( aerobic  AND workout )  OR  TITLE-ABS-KEY ( resistance  AND training )  OR  TITLE-ABS-KEY ( strength )  OR  TITLE-ABS-KEY ( strength  AND workout )  OR  TITLE-ABS-KEY ( circuit-based  AND exercise )  OR  TITLE-ABS-KEY ( combined  AND exercise )  OR  TITLE-ABS-KEY ( chronic  AND exercise )  OR  TITLE-ABS-KEY ( acute  AND exercise ) ) )  AND  ( ( TITLE-ABS-KEY ( control  AND groups )  OR  TITLE-ABS-KEY ( control  AND group* )  OR  TITLE-ABS-KEY ( volunteers )  OR  TITLE-ABS-KEY ( "not trained" )  OR  TITLE-ABS-KEY ( untrained )  OR  TITLE-ABS-KEY ( sedentary )  OR  TITLE-ABS-KEY ( unexercised ) ) )  AND  ( ( TITLE-ABS-KEY ( irisin )  OR  TITLE-ABS-KEY ( irisin  AND level )  OR  TITLE-ABS-KEY ( blood  AND irisin )  OR  TITLE-ABS-KEY ( plasma  AND irisin )  OR  TITLE-ABS-KEY ( serum  AND irisin )  OR  TITLE-ABS-KEY ( fndc5 ) ) )  AND  ( ( TITLE-ABS-KEY ( "clinical trials" )  OR  TITLE-ABS-KEY ( "randomized controlled trial" )  OR  TITLE-ABS-KEY ( "controlled clinical trial" )  OR  TITLE-ABS-KEY ( "clinical trial" )  OR  TITLE-ABS-KEY ( "randomized" )  OR  TITLE-ABS-KEY ( "placebo" )  OR  TITLE-ABS-KEY ( "drug therapy" )  OR  TITLE-ABS-KEY ( "randomly" )  OR  TITLE ( "trial" )  OR  TITLE-ABS ( "groups" )  OR  TITLE-ABS-KEY ( "intervention" )  OR  TITLE-ABS-KEY ( rct )  OR  TITLE-ABS-KEY ( non-randomized  AND controlled  AND trials )  OR  TITLE-ABS-KEY ( non-randomized )  OR  TITLE-ABS-KEY ( experimental  AND study )  OR  TITLE-ABS-KEY ( experimental )  OR  TITLE-ABS-KEY ( non-randomized  AND stud* ) ) ) ...View More | [137 document results](https://www.scopus.com/search/history/results.uri?origin=searchhistory&shid=6) |
| --- | --- | --- |
| 5 | ( TITLE-ABS-KEY ( "clinical trials" )  OR  TITLE-ABS-KEY ( "randomized controlled trial" )  OR  TITLE-ABS-KEY ( "controlled clinical trial" )  OR  TITLE-ABS-KEY ( "clinical trial" )  OR  TITLE-ABS-KEY ( "randomized" )  OR  TITLE-ABS-KEY ( "placebo" )  OR  TITLE-ABS-KEY ( "drug therapy" )  OR  TITLE-ABS-KEY ( "randomly" )  OR  TITLE ( "trial" )  OR  TITLE-ABS ( "groups" )  OR  TITLE-ABS-KEY ( "intervention" )  OR  TITLE-ABS-KEY ( rct )  OR  TITLE-ABS-KEY ( non-randomized  AND controlled  AND trials )  OR  TITLE-ABS-KEY ( non-randomized )  OR  TITLE-ABS-KEY ( experimental  AND study )  OR  TITLE-ABS-KEY ( experimental )  OR  TITLE-ABS-KEY ( non-randomized  AND stud* ) ) ...View More | [15,401,038 document results](https://www.scopus.com/search/history/results.uri?origin=searchhistory&shid=5) |
| 4 | ( TITLE-ABS-KEY ( irisin )  OR  TITLE-ABS-KEY ( irisin  AND level )  OR  TITLE-ABS-KEY ( blood  AND irisin )  OR  TITLE-ABS-KEY ( plasma  AND irisin )  OR  TITLE-ABS-KEY ( serum  AND irisin )  OR  TITLE-ABS-KEY ( fndc5 ) ) | [1,531 document results](https://www.scopus.com/search/history/results.uri?origin=searchhistory&shid=4) |
| 3 | ( TITLE-ABS-KEY ( control  AND groups )  OR  TITLE-ABS-KEY ( control  AND group* )  OR  TITLE-ABS-KEY ( volunteers )  OR  TITLE-ABS-KEY ( "not trained" )  OR  TITLE-ABS-KEY ( untrained )  OR  TITLE-ABS-KEY ( sedentary )  OR  TITLE-ABS-KEY ( unexercised ) ) | [2,027,962 document results](https://www.scopus.com/search/history/results.uri?origin=searchhistory&shid=3) |
| 2 | ( TITLE-ABS-KEY ( exercise )  OR  TITLE-ABS-KEY ( training )  OR  TITLE-ABS-KEY ( exercise  AND training )  OR  TITLE-ABS-KEY ( training  AND program )  OR  TITLE-ABS-KEY ( sport )  OR  TITLE-ABS-KEY ( sports )  OR  TITLE-ABS-KEY ( physical  AND activity )  OR  TITLE-ABS-KEY ( treadmill  AND exercise )  OR  TITLE-ABS-KEY ( physical  AND exercise )  OR  TITLE-ABS-KEY ( endurance  AND training )  OR  TITLE-ABS-KEY ( aerobic )  OR  TITLE-ABS-KEY ( aerobic  AND workout )  OR  TITLE-ABS-KEY ( resistance  AND training )  OR  TITLE-ABS-KEY ( strength )  OR  TITLE-ABS-KEY ( strength  AND workout )  OR  TITLE-ABS-KEY ( circuit-based  AND exercise )  OR  TITLE-ABS-KEY ( combined  AND exercise )  OR  TITLE-ABS-KEY ( chronic  AND exercise )  OR  TITLE-ABS-KEY ( acute  AND exercise ) ) | [4,005,349 document results](https://www.scopus.com/search/history/results.uri?origin=searchhistory&shid=2) |
| 1 | ( TITLE-ABS-KEY ( adult )  OR  TITLE-ABS-KEY ( adult* )  OR  TITLE-ABS-KEY ( adolescent )  OR  TITLE-ABS-KEY ( adolescent* )  OR  TITLE-ABS-KEY ( teenager* )  OR  TITLE-ABS-KEY ( humans )  OR  TITLE-ABS-KEY ( healthy  AND volunteers )  OR  TITLE-ABS-KEY ( healthy  AND people  AND programs )  OR  TITLE-ABS-KEY ( healthy  AND individuals )  OR  TITLE-ABS-KEY ( human  AND subject )  OR  TITLE-ABS-KEY ( healthy ) ) | [23,860,769 document results](https://www.scopus.com/search/history/results.uri?origin=searchhistory&shid=1) |
